# A case for characterizing declarative memory commission errors in healthy aging

**DOI:** 10.3389/fcogn.2024.1505492

**Published:** 2025-01-06

**Authors:** Ariana Popoviciu, Lauren L. Richmond

**Affiliations:** Department of Psychology, Stony Brook University, Stony Brook, NY, United States

**Keywords:** cognitive aging, declarative memory, commission errors, false memory, associative memory, hyper-binding

## Abstract

Cognitive psychologists typically characterize declarative memory performance in terms of omission errors, or information that is not reported at test. At the same time, there tends to be much less attention paid to characterizing errors of commission, such as reporting non-studied items at test. Importantly, older adults are known to make both types of errors in declarative memory tasks more often than young adults. This review aims to encourage a more thorough characterization of age-related commission errors in declarative memory research by synthesizing findings from disparate literatures that have taken an interest in characterizing this type of error. Specifically, findings relating to commission errors from the false memory, associative memory, and hyper-binding literatures are reviewed to demonstrate the utility of characterizing older adults' declarative memory performance by accounting for commission errors. Together, existing cognitive data provide a compelling rationale for memory researchers, and particularly those interested in age-related changes in memory, to characterize performance by accounting for commission errors in addition to the more commonly considered omission errors.

## Introduction

Cognitive aging is accompanied by both inter- and intra-individual changes in memory, but these changes are not uniform across memory systems. In fact, some memory systems, such as procedural memory or priming, exhibit relative preservation into late life, whereas other memory systems, such as some aspects of declarative memory, tend to show decline with age (Nyberg et al., 2012). One portion of declarative memory that exhibits significant deleterious effects of cognitive aging is episodic memory for specific past experiences (Nilsson, 2003; Ghisletta et al., 2012; Nyberg et al., 2012). Cross-sectional and longitudinal estimates tend to converge on the mid-fifties as the age at which such memory decline begins (Rönnlund et al., 2005; Nyberg et al., 2012; Glisky et al., 2022; Liampas et al., 2023). While age-related declines in episodic memory have been consistently reported (Cansino, 2009), performance decrements for older adults are typically the most pronounced in tasks that provide little environmental support, such as in free recall tasks, where participants are themselves responsible for both initiating and maintaining memory search to produce responses (Luo and Craik, 2009; Shing et al., 2010).

One convenient and frequently used metric to quantify the impact of aging on episodic memory task performance involves tallying the total number of correctly produced responses on a free recall task. In a typical free recall task, participants are presented with a list of items to study and recall, either immediately or after a delay. In one common neuropsychological test used to index episodic memory (the Rey Auditory Verbal Learning Test or RAVLT), for example, participants are presented with a list of 15 semantically unrelated words five times and attempt to recall the list immediately after each presentation (Rey, 1958). Next, participants are presented with a second 15-item word list to study and recall. Participants are then asked to recall the words from the first list again, and after an ~20-min delay participants complete delayed-recall and recognition tests for items from the first list. The delayed-recall portion is scored based on the number of correctly recalled items out of the total possible 15 items that the participant could have correctly recalled.

Although the RAVLT has adequate reliability and validity in older adult samples (de Paula et al., 2012; Hammers et al., 2022), there are some aspects of age-related changes in declarative memory that may not be well captured by characterizing performance by only accounting for correct items (as well as the inverse of this score, indicating omitted items). In addition to errors of omission, older adults may also make errors that involve incorrectly reporting non-studied information, known as commission errors. Importantly, commission errors can stem from a variety of declarative memory systems. For example, a participant may report the word “kitten” at test when the word that they actually studied was “cat,” potentially indicating reliance on a gist-based representation of information held in episodic memory. To take another example, this participant may also report the semantically related item “dog” at test; this sort of error is likely a result of interactions between information held in episodic and semantic memory given the semantic relationship between the words “cat” and “dog.” For this reason, we use the more neutral term “declarative memory” throughout when discussing past literature examining commission errors. In the section below, we briefly review theories of cognitive aging that are relevant to the production of commission errors.

## Relevant theories of cognitive aging

Many theories have been proposed to explain age-related changes in cognitive functioning. Prominent accounts in the cognitive aging literature include age-related decline in attention (Madden, 1986), inhibitory deficit theory (Hasher and Zacks, 1988), and overreliance on gist-based processing (Reyna and Brainerd, 1995). Though additional foundational theories in cognitive aging exist to explain age-related cognitive decline more generally, such as processing speed theory (Salthouse, 1996), the three accounts listed above are the most relevant to consider in the context of the unique features of commission errors in declarative memory tasks. Importantly, it is likely that these accounts may have both distinct and overlapping contributions to commission errors.

One notable hypothesis proposed to explain age-related changes in memory focuses on the idea that older adults have reduced attentional resources compared to young adults (Madden, 1986). These age-related attentional changes are most noticeable in complex attentional tasks, such as those that elicit selective attention or require divided attention (Commodari and Guarnera, 2008). Selective attention tasks require participants to sustain attention on one aspect of a task while ignoring unnecessary information. Divided attention refers to the ability to focus on more than one task at the same time. Though both selective and divided attention are impaired by advancing age (Murman, 2015), divided attention conditions are sometimes used in young adult samples to mimic the attentional resource limitation that this hypothesis suggests is the basis for age-related degradation in episodic memory. In addition to having more limited attentional resources, recent research suggests that older adults may have a wider attentional lens, where the information that enters selective attention is not as filtered as it is for young adults (Weeks and Hasher, 2018). Together, limited attentional resources and less selectivity for information entering one's attentional system could explain why older adults tend to perform more poorly on episodic memory tasks than young adults.

An alternative account offered by inhibitory deficit theory posits that memory decline emerges in old age due to older adults' reduced ability to inhibit goal-irrelevant stimuli, thoughts, and actions (Hasher and Zacks, 1988; Lustig et al., 2007; Weeks et al., 2020). Inhibition is thought to consist of access, deletion, and restraint components. “Access” refers to the purposeful limiting of attention to relevant aspects of the stimuli, similar to the description of selective attention provided above. One way in which this component has been tested is by asking participants to engage in a speed-based task while simultaneously being given distractors. For example, participants may be given a task in which pairs of letter strings are presented and are asked to decide whether the two letter strings are the same or different. At the same time, participants may be assigned to complete this task under a high-distraction condition where many pairs are presented on the screen or under low-distraction where just the two letter strings to be evaluated appear on screen (Lustig et al., 2006). Older adults are known to be uniquely slowed under high distraction compared to young adults, and older adults' performance is improved by the removal of such distraction (Lustig et al., 2007). The “deletion” component of inhibition refers to clearing away information from the mind that is no longer necessary, or was never needed (Campbell et al., 2020). In paradigms where participants are instructed to forget some information, older adults tend to report more of the “forget” information than young adults, indicating increased difficulty with recategorizing such information as irrelevant (Lustig et al., 2007). The “restraint” component refers to the ability to suppress inappropriate responses, which can complement the access feature of inhibition in successfully performing a task (Lustig et al., 2007). Restraint has been characterized in past research by asking participants to withhold a correct response to a task whenever a specific stimulus is presented. Here, age-related declines appear to be task-dependent, where some variations of these paradigms produce an age effect (e.g., go/no-go tasks), while others display age equivalence in performance (e.g., color-word Stroop task; Lustig et al., 2007; Campbell et al., 2020). Though there is some evidence of age-related decline in all three components of inhibition, the access function has been most consistently demonstrated as the basis for age-related declines in memory performance (Campbell et al., 2020), suggesting that a key deficit exhibited by older adults involves allowing irrelevant information to enter their cognitive systems during initial encoding.

Finally, a third hypothesis proposed to explain age-related memory decline suggests that older adults' overreliance on gist, or the general essence of information, rather than specific details when recalling episodic information (for a review, see Greene and Naveh-Benjamin, 2023) could result in poorer declarative memory performance in this age group. This idea is consistent with the tenets of fuzzy trace theory, which distinguishes between two different aspects of memory: verbatim representations, and fuzzy, or gist-based, impressions (Reyna and Brainerd, 1995). Research suggests that as individuals age, they increasingly depend on gist-based processing to compensate for declines in the ability to represent specific details, and this effect is especially pronounced at longer retention intervals (Greene and Naveh-Benjamin, 2022). Changes in the specificity of recalled information may be thought of as a complementary account to ideas focused on both reduced attention and inhibition in older adults. That is, it is possible that all of these age-related cognitive changes could negatively impact older adults' memory performance as well as give rise to increased commission error rates in aging.

## The omission-commission model

Theories regarding age-related changes in declarative memory reviewed above suggest that these deficits may emerge due to several different cognitive mechanisms, but they also lead to the suggestion that older adults may make both more omission and commission errors than cognitively normal young adults. Research from the everyday action literature has identified unique cognitive properties of such errors, advancing the idea that both error types are worth considering in studies of age-related changes in declarative memory.

The omission-commission model was first proposed in and applied to studies of everyday action as an alternative framework to resource theory, which suggests that action errors emerge as a result of general limitations in cognitive resources (Schwartz et al., 1998). Instead, the omission-commission model aimed to better capture different types of errors made in everyday action by different clinical populations (Giovannetti et al., 2002; Kessler et al., 2007; Giovannetti et al., 2008a). Omission errors in everyday action can simply be described as failures in performing a step in a task, analogous to the non-recalled items that are represented in the composite score of free recall tasks. With respect to commission errors, Giovannetti et al. characterize these as a missteps toward completing a task, which may take several forms, including action or object substitutions and action additions (Giovannetti et al., 2008a). In the context of a free recall task, substitutions may be thought of as comparable to reporting a semantically related or perceptually similar word while also omitting the presented word in a word-based free recall task, while additions may be akin to reporting a word at test that is unlike those presented during encoding. Although the bulk of the research on this model in aging has focused on individuals with Alzheimer's Disease (Giovannetti et al., 2002, 2008a), there is some evidence to suggest that healthy older adults produce more commission errors than omission errors (Giovannetti et al., 2008b; Bailey et al., 2013), and that poorer delayed free recall performance in healthy older adults predicts higher rates of “micro-errors,” considered to be a precursor to overt commission errors (Rycroft et al., 2018; Holmqvist et al., 2023). Together, these studies suggest that while omission errors provide valuable information about memory and aging, the cognitive basis for commission errors may be distinct from omission errors and may be more commonplace in healthy older adult samples than omission errors.

## Applying the omission-commission model to lab-based experimental memory tasks

Declarative memory research in cognitively normal older adults has primarily focused on omission errors (Rhodes et al., 2019) and there has been less interest in characterizing commission errors, particularly in the context of free recall tasks. Quantifying this error type in addition to omission errors is expected to allow for a more granular understanding of declarative memory change across the adult lifespan. Moreover, commission errors may also prove to have distinct underlying cognitive mechanisms from omission errors. Recognition memory tasks more readily lend themselves to characterizing this aspect of memory functioning via false alarms (Craik and McDowd, 1987; Isingrini et al., 1995). A large meta-analysis of over 200 studies focused on age differences in recognition memory suggests that older adults tend to adopt a more liberal response criterion than young adults, resulting in higher false alarm rates in older-aged samples (Fraundorf et al., 2019). At the same time, recognition memory tasks may not be as well-suited to capture more nuanced commission errors that older adults may make due to the high degree of environmental support afforded by the task itself. Despite free recall paradigms tending to elicit greater age-related differences in performance than recognition tasks (Craik and McDowd, 1987), characterizing age-related patterns in commission error rates in free recall remains an important and relatively underexplored area of research.

To make the importance of considering commission errors clear, let's take an example. Imagine two older adults who have the exact same overall score on a free recall task, both correctly recalling 11 out of 15 possible items. However, person A may have only listed out those exact 11 items, whereas person B may have reported 35 words in total, 11 of which appeared in the original list. Considering only their overall correct recall (11/15) or omission errors (4/15), these two older adults would be identical. However, their ways of getting to this score, and thereby their commission error rates, are vastly different. Specifically, person A would have committed 0 commission errors, whereas person B would have made 24 commission errors. Intuitively, on this basis, we would be inclined to say that person A performed better than person B. A key distinction that emerges between omission and commission memory errors in the context of this example are that omission errors are directly proportional to accurate recall, whereas commission errors can be independent of accurate recall. In other words, it is not possible to draw conclusions about older adults' commission errors just from their correct recall scores alone in the way that one could for omission errors. Extending this example to the real world, imagine a scenario where these two individuals represent items that they need to buy from the grocery store in declarative memory. Upon arriving, person B would be more likely to purchase several additional and unnecessary items at the grocery store than person A, resulting in a higher-than-needed grocery bill.

If we consider the pattern of findings from the everyday action literature relating to the omission-commission model, we may expect that commission errors could be a particularly sensitive indicator of age-related declarative memory impairment. In spite of the more limited research on commission errors compared to omission errors in the context of lab-based experimental memory tasks, this topic has received attention specifically within the false memory literature via the Deese-Roediger-McDermott (DRM) paradigm (Deese, 1959; Roediger and McDermott, 1995), as well as the associative memory and hyper-binding literatures. Moreover, such research often focuses on age-related differences in these processes. To foreshadow, these bodies of work suggest that there is much to be learned in the broader literature focused on experimental lab-based memory tasks by characterizing commission errors, and that doing so will allow researchers to obtain a more comprehensive understanding of older adults' declarative memory function. This can be achieved not only through the creation of new paradigms that evoke commission errors, but also by mining extant data to examine patterns in the way people respond on commonly used tasks when such data are available. The following review of research on commission errors in the DRM, associative memory, and hyper-binding literatures highlights the unique insights about age-related changes in declarative memory function that can be gleaned via careful consideration and quantification of commission errors.

## Commission errors in false memory: a review of findings from the Deese-Roediger-McDermott paradigm

**Figure 1:**
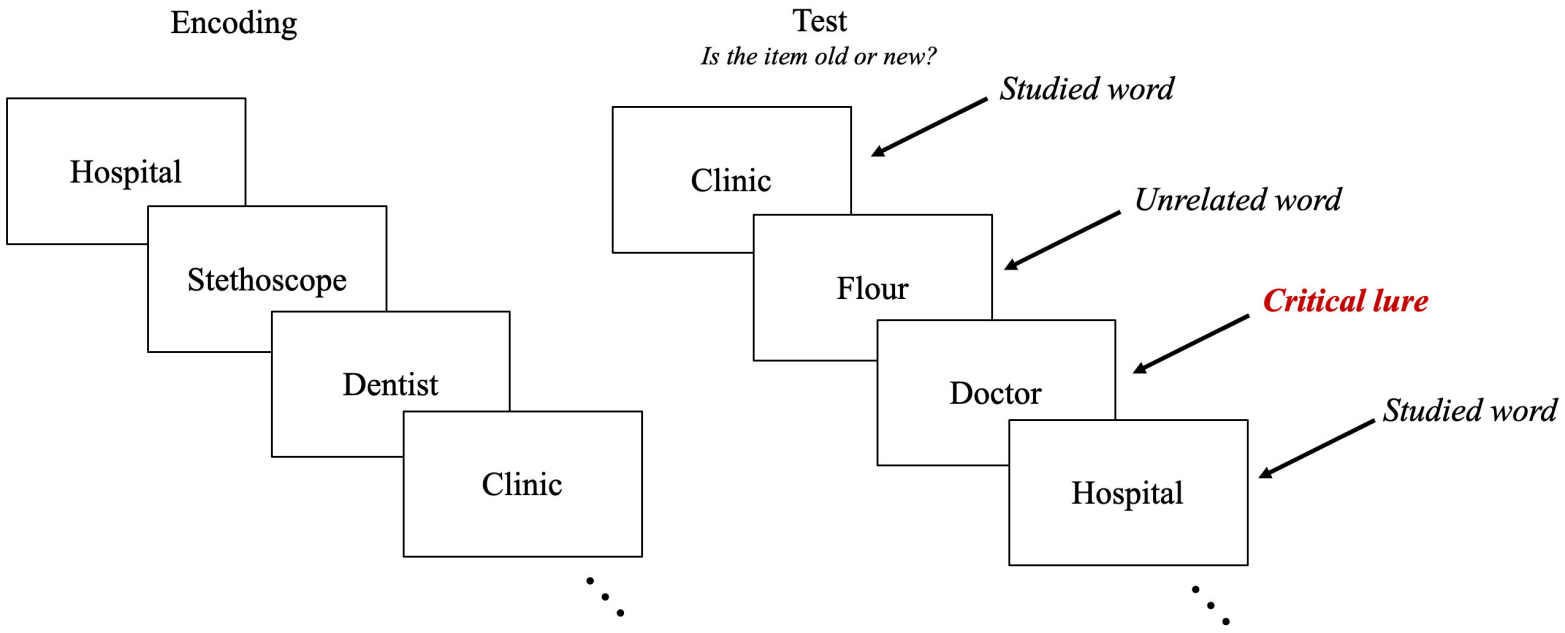
DRM experimental paradigm. Study list is semantically related. Participants are given the opportunity to recall words from memory (i.e., free recall) prior to the recognition task.

The cognitive behavioral literature on false memory, or memory for information that was not presented, necessarily focuses on errors of commission. Though it is not the goal of this paper to provide a comprehensive overview of the active theoretical debates in the false memory literature, it is worth delving into some specific evidence from this perspective around the role of declarative memory commission errors in older adults. Importantly, this paper focuses on evidence regarding commission errors produced in the context of a common experimental laboratory-based false memory paradigm, the DRM task (Deese, 1959; Roediger and McDermott, 1995, see Figure 1). Participants in this paradigm are presented with a list of words that are all semantically related to one another and also relate to a critical, but not presented, word. After being presented the list, memory for the items contained in the earlier-presented list is tested. For example, participants might be presented with the words *nap, snooze, pillow*, and *bed* which all relate to the unpresented critical lure *sleep*. At test, participants are very likely to produce (in free recall tasks) or endorse (in recognition tasks) the critical lure as being part of the original study list (Pardilla-Delgado and Payne, 2017). Of note, the errors produced via free recall in the DRM paradigm are entirely self-generated. This phenomenon likely relates closely to source monitoring difficulties, whereby the source of a particular piece of information is incorrectly attributed (Johnson, 1997). That is, DRM commission errors stem from the participant themselves activating their internal representation of the critical lure and later reporting that critical lure at test, incorrectly attributing the source of the information as the initial study list.

Source memory difficulties are common with increasing age (Brown et al., 1995; Glisky et al., 2001). It is therefore unsurprising that older adults are also particularly susceptible to recalling or endorsing critical lures in DRM paradigms, and that they display relatively high confidence when they do so (Devitt and Schacter, 2016; Abichou et al., 2022). These patterns have been observed for a variety of different types of DRM stimuli, including word lists (see e.g., Norman and Schacter, 1997; McKelvie, 2004; Smith et al., 2005; Thomas and McDaniel, 2013), images (Gallo et al., 2009), and names of famous people (Plancher et al., 2009). Interestingly, even when given warnings about the potential for false memories to emerge in this task, critical lure recall is not significantly reduced in older adults (McCabe and Smith, 2002; Watson et al., 2004).

One reason why older adults may be particularly susceptible to producing false memory commission errors is due to the role of limited attentional resources on false memory performance. Under conditions of divided attention, young adults display increased false memory rates that are comparable to those observed in older adults (Skinner and Fernandes, 2009). Divided attention conditions have also been used to distinguish among young and older adults' DRM commission error rates when participants are given the opportunity for repeated study. Under full attention, young adults' memory performance tends to benefit from multiple study opportunities, whereas older adults under full attention and young adults under divided attention show increases in false memory with repeated study (Jacoby, 1999; Kensinger and Schacter, 1999). In addition to the contributions of reduced attentional capacity to older adults' false memory recall, it is further believed that increased false memory rates in older adults may reflect older adults' greater reliance on gist-based memory representations (Dennis et al., 2014) and difficulties with the encoding and/or retrieval of specific details (Duarte et al., 2010), which together serve to increase the likelihood of false recall. As an example, when older adults are asked to engage in distinctive processing for items on the DRM paradigm, their ability to correctly identify items from the study list is increased and their susceptibility to endorsing critical lures is reduced (Huff and Aschenbrenner, 2018). These findings are consistent with fuzzy trace theory, which is often offered as an explanation for false memory (Reyna and Brainerd, 1995; McKelvie, 2004). Finally, the false memory literature offers a clear link to the contributions of reduced inhibition in older adults. In a study where older adults were categorized as having high or low levels of inhibitory skill based on their performance on a battery of inhibition tasks, those in the lower inhibitory skill group produced significantly more false memories and also exhibited fewer correct recalls compared to those classified as having high inhibitory skill (Colombel et al., 2016). The increased false memory rate in the low inhibitory skill group may reflect disturbances in restraint inhibition, or difficulty with suppressing an incorrect response, and may also align with higher susceptibility to source memory errors.

Together, evidence from the DRM paradigm suggests that false memory commission error rates increase in older adulthood, where older adults are likely to produce critical lure items with high confidence. The increased critical lure rates exhibited by older adults may be related to some of the more general cognitive changes that are known to occur in aging, including reduced attentional resources, inhibitory deficits, and increased gist based-processing.

## Commission errors in associative memory

Information in memory can be organized by forming associations between previously unrelated items (Anderson and Bower, 2014), a process known as associative memory. Associative memory allows for the integration of information and the retrieval of complex relationships between stimuli. Because information encountered in the real world often benefits from the formation of associations between previously unrelated pieces of information, such as associating a location in one's household with their keys, associative memory is a key component of everyday memory functioning (Koller and Cannon, 2023).

**Figure 2:**
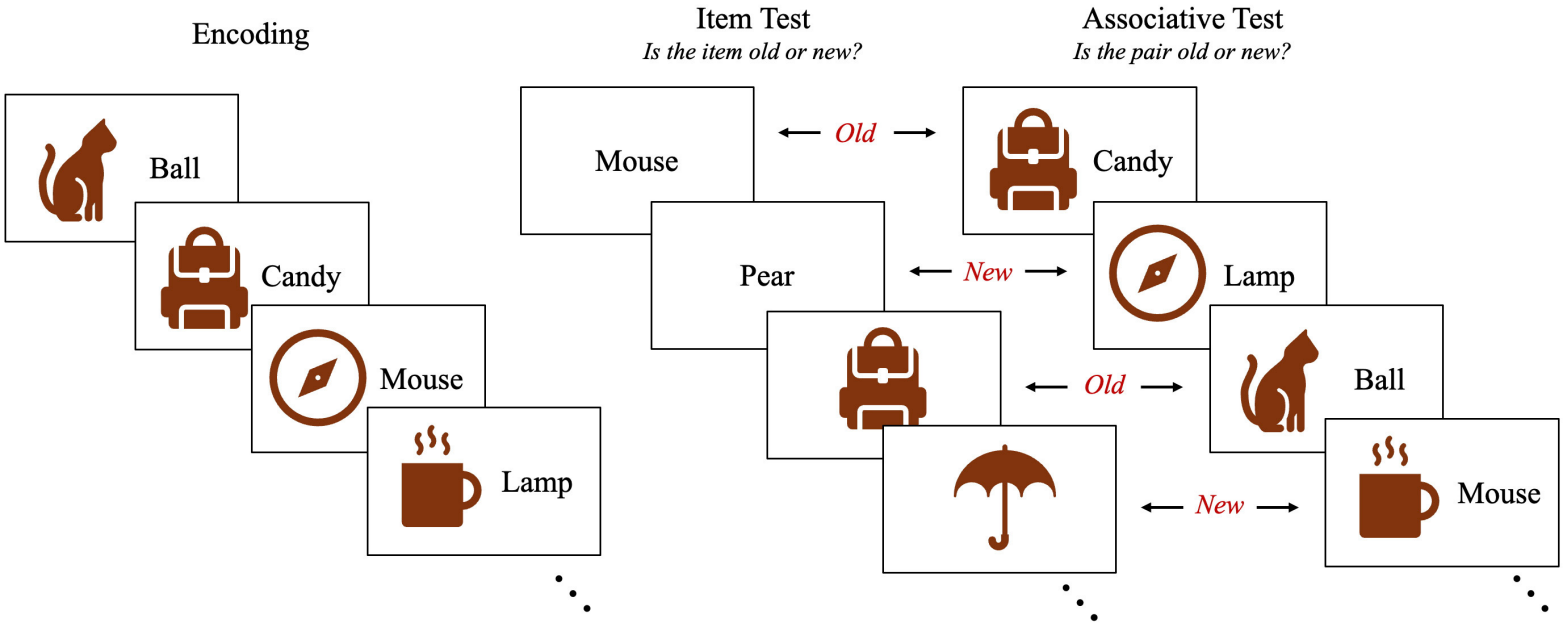
Associative memory experimental paradigm. Item tests are usually given individually such that participants are tested on one feature at a time (e.g., word list first, then picture list).

At the same time, older adults exhibit deficits in associative memory. That is, older adults struggle with creating and retrieving associations between unrelated pieces of information (Naveh-Benjamin, 2000; Naveh-Benjamin and Mayr, 2018). Associative memory is often tested using a variant of a paired-associates task (Naveh-Benjamin, 2000; see Figure 2). For example, using stimuli consisting of words paired with photos, participants are asked to study either one of these two features (the word or the photo), or they are asked to study both features in tandem, forming an association. After a distractor phase, participants are tested on their memory for both the individual aspects, termed item memory, as well as their associations (i.e., associative memory; Naveh-Benjamin et al., 2003). Regardless of the initial study conditions, older adults tend to perform comparably to young adults on tests of item memory, where they are asked to report their memory for the individual features rather than the relationships between features. Conversely, older adult performance tends to be significantly worse than young adult performance on tests of associative memory (Naveh-Benjamin, 2000; Naveh-Benjamin et al., 2003, 2004b; Old and Naveh-Benjamin, 2008a).

Compared to commission errors produced in the context of the DRM wherein the participant internally generates the critical lure item, commission errors in associative memory tasks can be produced when a participant remembers the item stimuli presented in the context of the experiment but incorrectly remembers how items were paired together. For example, older adults may be inclined to accept incorrectly paired items as being studied in the encoding phase. Of course, it is also possible to produce commission errors for items that were not presented earlier. While some work attributes older adults' poorer performance on associative memory tasks to older participants producing both lower hit rates and higher false alarm rates (Bender et al., 2010), other work suggests that this effect is mainly attributable to increased false alarm rates exhibited by older adult samples (Naveh-Benjamin et al., 2009). Increased false alarms in associative memory with age are of particular interest to the discussion of commission errors. It is acknowledged that in cases where the associative memory deficit is derived from a mix of lower hit rates and higher false alarms, it is more difficult to detangle omission errors from commission errors. Many studies on the associative memory deficit do not separately analyze hit rates and false alarms, further adding to the difficulty of strictly isolating commission errors. Nevertheless, although it is likely that both types of errors contribute to the associative memory deficit (Bender et al., 2010), some prior evidence suggests that false alarms (i.e., commission errors) may be the driving factor for age differences in performance (Naveh-Benjamin et al., 2009).

A meta-analysis of 90 studies affirmed that older adults display greater deficits in associative memory than in item memory compared to young adults (Old and Naveh-Benjamin, 2008a). Of note, there is a significant positive correlation between item and associative memory performance, such that those participants who display better item memory also tend to display better associative memory regardless of the age group to which they belong (Old and Naveh-Benjamin, 2008a). Moreover, the few studies within this meta-analysis that have found larger effects of age on item memory compared to associative memory nonetheless report noticeable age effects in associative memory (e.g., Mather and Johnson, 2000; Bastin and Linden, 2005). On the whole, older adults display a general disadvantage in associative memory, and when item memory is impaired, associative memory performance is expected to be poorer still.

These findings extend across a variety of study designs, including being evident in tests of source memory, temporal order memory, spatial location memory, word-word pairings, picture-word pairings, videoclips, and in both verbal and non-verbal memory tests (Naveh-Benjamin et al., 2004b; Old and Naveh-Benjamin, 2008a,b). Associative source memory is typically tested by having participants learn associations between specific words and the voice that presented the item. Associative temporal order memory tests focus on the sequential associations of items presented across time, or the identification of which block an item was presented in. Studies of spatial location memory examine participants' ability to form relationships between where on a screen an item was presented, while word-pairing studies test associations between unrelated word items (Old and Naveh-Benjamin, 2008a). Further evidence of an age-related associative memory deficit has been found when testing associative memory for words superimposed on pictures of faces (Naveh-Benjamin et al., 2004a, 2009) and dynamic videoclips of different individuals completing specific actions (Old and Naveh-Benjamin, 2008b).

Older adults have significantly higher false alarm rates than young adults on associative memory tests when the encoding instructions are intentional, meaning that they are told about upcoming tests before studying. The age-related associative memory deficit is diminished, though still present, when encoding instructions make no mention of the true purpose of the study (Old and Naveh-Benjamin, 2008a; Naveh-Benjamin et al., 2009). Importantly, young adults perform similarly regardless of whether they were provided with intentional and incidental encoding instructions (Old and Naveh-Benjamin, 2008a; Naveh-Benjamin et al., 2009). Moreover, in studies comparing the effect of intentional and incidental learning on associative memory performance, no significant differences in age-related effect sizes were observed for item memory tests, indicating that the effect in the intentional encoding condition was mainly attributable to a heightened associative memory deficit for older adults (Old and Naveh-Benjamin, 2008a). Since participants were made aware of the need to study associative information for the upcoming test but were not given specific instructions about how to do so, one possibility to explain the age-related associative memory deficit observed across studies is that older adults have greater difficulty with self-initiating the processes necessary to form associations between units of information (Craik, 1986; Old and Naveh-Benjamin, 2008a). In line with this hypothesis, supplying older adults with the strategy of incorporating word pairs into sentences at encoding, and especially at encoding and retrieval, has been shown to reduce older adults' associative memory deficit relative to young adults (Naveh-Benjamin et al., 2007). At the same time, even when older adults are able to achieve verbatim recollection of a sentence they created to help solidify associations between word pairs, they still exhibit poorer recall of the associated target word than young adults (Hertzog et al., 2013).

The age-related associative memory deficit is most pronounced during older adults' less preferred time of day, namely the evening. In addition, those participants who report greater subjective memory complaints also have greater difficulty on associative memory tasks (Naveh-Benjamin and Mayr, 2018). In general, older adults are known to have lower confidence than young adults in their responses as well as poorer calibration between confidence and accuracy. As a result, if older adults are given the option to retrieve a word pair from declarative memory or, alternatively, use a visual probe to support retrieval, older adults tend to choose to use the visual probe, a pattern that has been termed *retrieval reluctance* (Hertzog and Touron, 2011). Moreover, study instructions that aim to either elicit or eliminate age-related stereotype threat can modify the intensity of difficulties with associative memory, with conditions that attenuate threat resulting in a smaller deficit (Brubaker and Naveh-Benjamin, 2018). In a similar vein, reliance on semantic support, such as evoking schematic knowledge about the environment, can benefit older adults' performance on associative memory tasks (Naveh-Benjamin et al., 2003; Naveh-Benjamin and Mayr, 2018).

Another important consideration with respect to age differences is how specific the details in question that are being tested. Older adults' performance tends to suffer when the task requires the recollection of very granular details, but they perform similarly to young adults when asked to retrieve information that is less granular (Greene and Naveh-Benjamin, 2020), consistent with an increased reliance on gist-based processing in older adults. This has been tested by pairing faces with scenes (e.g., park, mall, kitchen, desert) under intentional learning conditions, and later testing their memory by displaying intact, related, and unrelated probes. Older adults exhibited similar associative memory scores to young adults when they were asked about broad scene categories (e.g., “Was this face indoors?”). However, older adult performance declined relative to young adults when the categories were narrowed down (e.g., “Was this face in *a* kitchen?”) and was further impaired when tested on highly specific associations (e.g., “Was this face in *this* kitchen?”). Though this study does not directly address whether these specificity-related associative memory deficits emerge due to processing at encoding or retrieval, it is likely a combination of the two (Greene and Naveh-Benjamin, 2020).

The question of whether associative deficits in older adults emerge during encoding or retrieval has been a major area of study. Some have proposed that older adults are less able to encode associations due to a reduction in available attentional resources (Naveh-Benjamin and Mayr, 2018). To test the idea that attention is necessary for associative memory, studies have experimentally manipulated young adults' available attentional resources by placing them under divided attention conditions in an effort to more closely resemble older adults' available attentional resources. One commonly used divided attention task in studies of associative memory involves the auditory presentation of numbers to participants and having them tap a button whenever a series of odd numbers are presented in sequence (Castel and Craik, 2003; Craik et al., 2010; Cooper and Odegard, 2011). Using this paradigm, conflicting evidence for the role of attentional resources in associative memory has emerged. While some work has observed the associative memory deficit in young adults under divided attention (Castel and Craik, 2003), other studies have failed to find comparable patterns of associative memory performance in older adults generally and young adults under divided attention (Naveh-Benjamin et al., 2004a,b; Kilb and Naveh-Benjamin, 2007; Craik et al., 2010; Cooper and Odegard, 2011). Specifically, while full-attention older adults display a distinct associative memory deficit, dividing the attention of young adults at encoding has been found to result in deficits for both item and associative memory, while placing young adults under a divided attention condition at test does not result in any deficits relative to full attention conditions (Craik et al., 2010; Cooper and Odegard, 2011). Given that the role of attention in associative memory has primarily been examined by using divided attention paradigms in young adult participants, it is possible that this manipulation does not offer an accurate simulation of attention in older adults, therefore limiting the conclusions about the role of attention in associative memory that can be drawn from such approaches.

Another proposed mechanism for the associative deficit in aging is reduced unitization (Naveh-Benjamin and Mayr, 2018), or the ability to represent multiple individual items as a single unit (Graf and Schacter, 1989). This can be achieved through a variety of mechanisms, such as semantic binding (Patterson et al., 2009), or visually embedding features of stimuli (Bastin et al., 2013). Unitization is known to enhance performance on associative memory tests, but not item memory tests, in young adults (Parks and Yonelinas, 2015). This effect has been attributed to reducing the cognitive demand needed to successfully complete the task, as well as minimizing the reliance on the hippocampus (Haskins et al., 2008). By extension, since older adults typically possess more limited cognitive resources and display reduced hippocampal volumes, it is likely that they would benefit greatly from increased unitization. Research with both older adults and younger adults corroborating this speculation shows that unitization at encoding improves older adult performance on associative memory by visually superimposing images (Overman et al., 2018), or by having the target features of the independent stimuli interact (Bastin et al., 2013). Another study that aimed to distinguish the effect of unitization strategies among older adults at a more granular level investigated whether motion of items, fusion of items, having one item act on another (action/consequence), or the combination of the three impacts associative memory. Older adult performance was improved in the action/consequence condition and in the condition that combined all three components of unitization relative to the other unitization strategies tested (D'Angelo et al., 2017). These data suggest that although spontaneous use of unitization tends to be reduced in older adults (Patterson et al., 2009), unitization can be a useful strategy to improve associative memory performance in older-aged samples.

At retrieval, it has been suggested that older adults may engage in less strategic recollection than young adults (Naveh-Benjamin and Mayr, 2018). This claim comes from a variety of different associative memory studies that test how well older adults control their retrieval processes. For example, specificity-related deficits in associative memory (Greene and Naveh-Benjamin, 2020) have been reported, suggesting that older adults' associative memory performance suffers most on tests that require the retrieval of highly specific details (Luo and Craik, 2009), related to the hypothesis on increased gist-based processing. Interestingly, manipulations intended to either improve or disrupt encoding did not interact with the specificity effect at retrieval (Luo and Craik, 2009). In a related paradigm that placed high or low demands on self-initiated associative memory retrieval, older adults performed worse in the high demand condition, providing evidence for impaired retrieval over and above impaired binding of information at encoding (Cohn et al., 2008). Use of strategy has also been probed via noun-pair retrieval shift, where individuals have the opportunity to switch from visual search to associative memory in order to more efficiently identify a pair of probe words among a list of several pairs. Older adults are more resistant than young adults to adopt this strategy shift, instead opting to continue verifying their answers with visual search (Touron and Hertzog, 2004; Hertzog and Touron, 2011). Further support for less strategic retrieval among older adults comes from the use of semantic knowledge in associative memory tasks (Naveh-Benjamin et al., 2003; Naveh-Benjamin and Mayr, 2018). In addition to conditions that are either consistent or inconsistent with prior knowledge, one study also manipulated the time allowed to complete the task, which was intended to differentially impact the engagement of cognitive control (Amer et al., 2018). Findings revealed that for information that was inconsistent with prior knowledge, age differences in performance favoring young adults emerged only in the slow condition; performance was similarly poor between age groups in the fast condition. The authors suggested that reductions in strategic controlled retrieval in older adults contributed to their reduced ability to form arbitrary associations even when given enough time to form such associations. Conversely, young adults were able to take advantage of the increased time allotted to them in the slow condition to bolster their associative memory performance (Amer et al., 2018).

Another retrieval-based explanation that has been offered for the age-related associative deficit is that older adults have more difficulty with reinstating of the organization of the initial stimuli, making it more difficult to access the associations that link items together (Naveh-Benjamin and Mayr, 2018). One study that tested how the associative memory deficit may be impacted by different types of associations presented participants with stimuli consisting of faces and scenes that were either superimposed (labeled the item-context condition) or presented side by side (labeled the item-item condition). The organization of the stimuli was further manipulated at retrieval by presenting the stimuli for recognition either in the same configuration as the encoding phase (congruent) or the opposing configuration (incongruent). As the researchers hypothesized, older adults struggled with recalling associations when the presentation of items at test was incongruent with how they were presented at encoding, though this finding only emerged in the item-context condition. No such deficit was observed in the young adult sample. These findings suggest that older adults' associative memory performance benefitted from encoding-retrieval congruency (Overman et al., 2018) consistent with key tenets of the environmental support hypothesis (Craik, 1986).

The combination of less strategic recollection and difficulty reinstating stimulus organization in older-aged samples has been tested through the use of targeted instructions during a paired-associates task that randomly assigns participants to generate a sentence or an interactive image incorporating both unrelated words (Hertzog et al., 2013). Despite similar characteristics in the generated mediators between age groups at encoding, older adults scored more poorly than young adults on several dimensions related to the recollection of sentence or image mediator, as well as on recalling the associated target. Specifically, older adults were less likely to retrieve mediators, particularly sentence mediators, and when they did, their descriptions relied more heavily on gist-based recall. This study suggests that a reliance on gist-based representations increases the likelihood of activating alternative, though incorrect, target words. This is consistent with the finding that older adults showed numerically more commission errors in the form of reporting an incorrect mediator (Hertzog et al., 2013).

Together, extant research suggests that a variety of factors at both encoding and retrieval may contribute to the associative memory deficit exhibited by older adults. This effect is thought to emerge not only because older adults may miss endorsing correct answers (i.e., omission errors) but also, and perhaps more often, endorse incorrectly paired items (i.e., commission errors). There is also a wealth of evidence to suggest that experimental and environmental features alike can influence the degree to which older adults exhibit associative memory deficits. At the same time, the age-related associative memory deficit appears to be difficult to eradicate completely. Returning to theories of cognitive aging, the associative memory deficit exhibited by older adults appears to be related to difficulties with reduced attentional resources and increased gist-based processing. Less support is found for inhibitory deficits in older adults explaining age-related difficulty with associative memory tasks.

## Commission errors in hyper-binding

**Figure 3:**
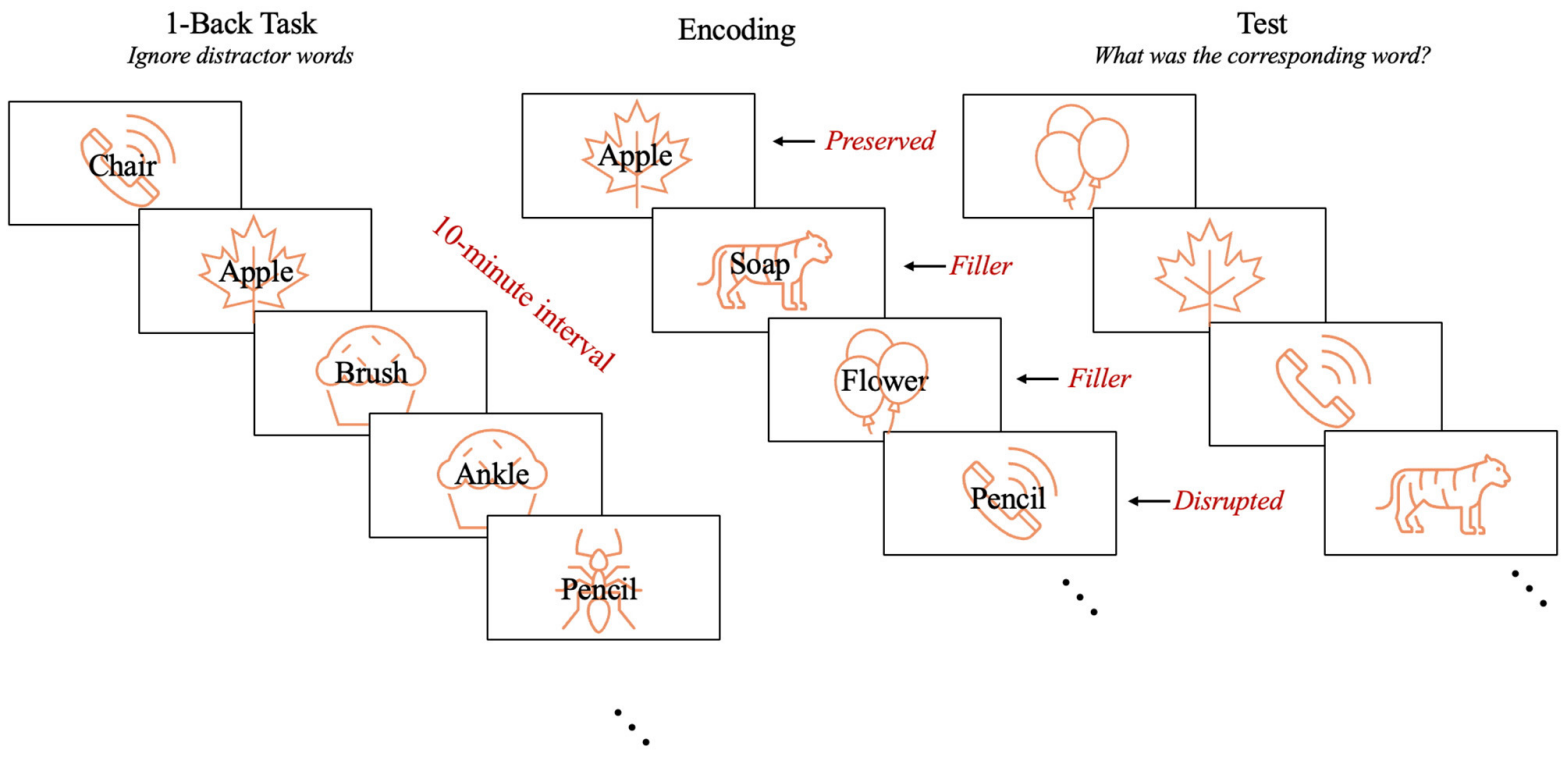
Hyper-binding experimental paradigm. Repeat pairs from the 1-Back task are not repeated in the latter portion of hyper-binding experimental designs.

A memory phenomenon that is related, though distinct, from associative memory is hyper-binding. Hyper-binding refers to the encoding extra, unnecessary information from the environment, which carries over into subsequent tasks, and is commonly observed in cognitively normal older adult samples (Campbell et al., 2010; Thomas and Hasher, 2012; Biss et al., 2013; Weeks et al., 2016; Davis et al., 2021). Commission errors in the hyper-binding literature are thought to arise from older adults having difficulties in inhibiting retrieval of no-longer-relevant associations. The most common paradigm used to test hyper-binding is a variant of a paired-associates task somewhat similar to paradigms reported in the associative memory literature (see Figure 3). In studies of hyper-binding, participants typically first engage in a 1-back task where they are presented with a series of pictures with words superimposed. Participants are instructed to ignore the words while paying attention to the pictures and to press a button when the same picture appears consecutively. Following a distractor period, participants then study another list of picture-word pairs and are instructed to memorize the pairings. This list includes both preserved pairs, meaning that they were repeated from the portion of the task before the distractor, and disrupted pairs, which consists of items (i.e., pictures and words) that are rearranged into novel pairings. No mention is made of the relation to the initial phase of the task. Finally, in the test phase, participants are presented with pictures and asked to recall the corresponding words from the most recent study phase. Overall, older adults tend to recall fewer words than young adults. However, while young adults show no difference between performance on disrupted and preserved picture-word pairs, older adults exhibit a unique advantage for remembering preserved pairs relative to disrupted pairs. That is, the difference score for preserved—disrupted pairs is typically larger in older adults in spite of all participants being instructed to ignore the word information presented during the initial 1-back task (Campbell et al., 2010). Here, commission “errors” emerge from older adults' endorsement of preserved pairs at a higher rate than disrupted pairs. Because preserved pairs were presented in the context of the earlier 1-back task, stimuli leading to commission errors are externally generated. Unlike commission errors in the DRM paradigm, which involves the internal generation of the critical lure item, and associative memory commission errors which seem to stem from a combination of external sources and internally-generated information, hyper-binding tasks necessarily expose participants to the information that can lead to commission made at a later point in the task.

The magnitude of the hyper-binding effect may be dampened or pronounced based on the parameters of the task. Older adults do not tend to show evidence of hyper-binding when they are given motivational incentives, such as points earned for correct responses, regardless of whether the incentive is of high or low value (Swirsky and Spaniol, 2020). In addition, the magnitude of the hyper-binding effect depends on whether task instructions are made explicit or left implicit. Participants can either be made aware of the connection between information presented during the 1-back task and the stimuli appearing in the later study/test phase (explicit), or may be left unaware of the relationship (implicit; Campbell and Hasher, 2018). The earliest version of the hyper-binding effect found by Campbell et al. (2010) was solely examined under implicit conditions. This explicit/implicit distinction was later found to have no effect on young adults' performance, but older adults exhibited a smaller performance difference for preserved—disrupted pairs in the explicit condition, which essentially eliminated the hyper-binding effect (Campbell and Hasher, 2018).

As in the associative memory literature, time of day effects have been observed for hyper-binding. Older adults are less able to ignore irrelevant information during their less preferred time of day, toward the evening, which may be related to attentional resources being depleted during non-preferred time of day (Anderson et al., 2014). To test the role of attention in hyper-binding directly, researchers examined the possibility that individual differences in attentional control in young adults may impact the magnitude of hyper-binding, with the idea that young adults with low attentional control would perform similarly to older adults. As expected, on average, young adults did not show overall evidence of hyper-binding. However, when examining performance in young adults with poor attentional control, a stronger hyper-binding effect emerged compared to young adults with good attentional control. The authors suggested that this pattern was likely related to lower inhibition of distracting information in young adults with poor attentional control (Davis et al., 2024). Finally, hyper-binding in older adults is heightened when items are close together temporally in the study list (Campbell et al., 2014). The authors suggest that learning lists of word pairs, as was done in this study, necessitates limited and directed attention. Difficulties with information suppression and deletion, as is common in older adults (Lustig et al., 2001; Campbell et al., 2020), allows for words presented in close proximity to one another to be unintentionally bound together (Campbell et al., 2014).

At the surface level, the hyper-binding effect seems to directly contradict the age-related associative memory deficit described above. However, the effect of task instruction and the addition of the 1-back task seems to influence whether older adults display performance patterns consistent with hyper-binding vs. impaired associative memory. Returning to studies that suggest a lack of hyper-binding when older adults are made aware of the initial study phase and the information is tagged as relevant (i.e., the explicit condition), this design is much more similar to the paradigms used to test associative memory (Campbell and Hasher, 2018; Davis et al., 2021). Additionally, findings from early associative memory experiments overlapped in some important ways with research on hyper-binding (Naveh-Benjamin, 2000). When young and older adult participants were asked to study one aspect of a word-font pairing and were then tested on the opposing feature (e.g., studying the font and then taking a word test), a numerical trend in performance favoring older adults was observed (Naveh-Benjamin, 2000). This pattern suggests that older adults were more likely than young adults to encode seemingly irrelevant information, although the effect observed in this study was not as pronounced as in true hyper-binding experiments.

Another study which has tried to better understand the relationship between the hyper-binding effect and associative memory deficit, albeit in working memory, supports the notion that a key design feature that distinguishes these two phenomena consists of whether the information is deemed relevant or irrelevant for later use (McCormick-Huhn et al., 2018). Specifically, McCormick-Huhn et al. (2018) found that performance patterns consistent with an associative memory deficit emerges when information is deemed relevant. The findings were less conclusive overall with respect to the role of relevance in producing a hyper-binding effect, in part due to differences in the paradigm used in this study compared to designs that more typically elicit hyper-binding. In an interesting twist, researchers have also tried to make use of the hyper-binding phenomenon to improve face-name associative memory by offering names as a distractor rather than as explicit features (Biss et al., 2018). Younger and older adults engaged in a task where they were presented with face-name pairs and then completed both immediate and delayed memory tests. During the delay period, participants also completed a seemingly unrelated face judgement task where a subset of the original pairs reoccurred as distractors, thereby providing re-exposure to these preserved pairs. In this context, only older adults exhibited improved recollection performance and less forgetting for the repeated pairs compared to the unrepeated ones (Biss et al., 2018). Altogether, it seems that older adults have a hard time not using connections between items that are picked up unintentionally to drive later task performance, which is distinct from their deficits in forming intentional associations between stimuli. It may therefore be useful to think of age-related increases in hyper-binding and deficits in associative memory as complementary, rather than contradictory, memory phenomena that emerge under different testing conditions and are perhaps influenced by participants' beliefs about the later relevance of information.

## Neurobiological contributions

Neuroimaging research has, by-and-large, also focused on errors of omission in declarative memory. Past work in this vein has provided evidence that although whole-brain volume declines with age, some regions of the brain implicated in declarative memory function (Simons and Spiers, 2003; Diana et al., 2007; Anand and Dhikav, 2012; Fan et al., 2017; Epelbaum et al., 2018) show accelerated age-related volumetric decline including in the frontal and temporal cortices as well as the hippocampus (Scahill et al., 2003; Fjell et al., 2009; Fujita et al., 2023). However, data from the everyday action literature suggests that there are both overlapping and unique neural substrates associated with omission and commission errors respectively. In a study involving patients with dementia, higher omission error rates in everyday action were found to be related to lower hippocampal volumes, while greater commission errors rates were associated with a broader host of structural brain markers including lower white matter volume, cortical gray matter volume, as well as hippocampal volume (Seidel et al., 2013). A similar imaging study that included a sample of cognitively normal older adults in addition to those with mild dementia symptoms also supports the idea that omission and commission errors are related to distinct neural substrates (Bailey et al., 2013). Of key interest to healthy aging, commission errors taking the shape of action additions were uniquely predicted by anterior cingulate cortex (ACC) volume, and remained significant after controlling for dementia severity (Bailey et al., 2013). Though much of the research that has distinguished omission and commission errors comes from pathological aging samples, these data nonetheless suggest that the neural substrates of omission and commission errors differ. Further distinguishing the neurobiological underpinnings of omission and commission errors in samples of cognitively normal older adults is a fruitful avenue for future research.

Within each of the literatures reviewed above, age differences in the activation patterns exhibited by young and older adults in response to the DRM task (Dennis et al., 2008, 2014; Kurkela and Dennis, 2016), associative memory tasks (Sperling et al., 2003; Miller et al., 2008; Zamboni et al., 2013; Bauer et al., 2015; Becker et al., 2015), and hyper-binding tasks (Campbell et al., 2012; James et al., 2016; Weeks et al., 2020) have been reported. Deactivation of the default mode network (DMN) has been shown to support task performance (Lustig et al., 2003), and older adults are known to exhibit reduced DMN deactivation during task challenge relative to young adults (Persson et al., 2007). Therefore, future studies of commission errors more generally may focus on DMN activity as a functional correlate of commission errors due to its relation with attention, inhibition, and off-task processing (Gusnard and Raichle, 2001; Lustig et al., 2003; Persson et al., 2007; Park et al., 2013). These interesting points of possible convergence with other neurobiological signatures of aging strengthen the need for a comprehensive understanding of how different error types are impacted by neurobiological changes in the substrates of declarative memory and functional changes in DMN activity with age.

## Conclusions and future directions

Reflecting on the false memory, associative memory, and hyper-binding literatures, common themes relating to foundational theories of cognitive aging have emerged that may help to explain commission errors in declarative memory more broadly. Increased attentional deficits with age have shown the potential to contribute to commission errors across all three literatures, including increases in false memory, difficulty binding relevant information together under explicit conditions, and unintentionally binding irrelevant information under implicit conditions (Skinner and Fernandes, 2009; Tsang, 2013; Davis et al., 2024). Inhibition is also frequently described as a relevant cognitive process in these literatures, and inhibitory skill is known to decline with age and can result in poorer declarative memory performance (Lövdén, 2003). More specifically, inhibitory deficit theory relates to false memory susceptibility in older adults and is central to the hyper-binding effect (Campbell et al., 2010). At the same time, age-related inhibitory deficits appear to be, at best, a minor player in age-related associative memory deficits (Guez and Naveh-Benjamin, 2015; Tanberg et al., 2022). Finally, support for older adults' overreliance on gist-based processing or information held in semantic memory is at the very center of the false memory literature, and the associative memory literature has also shown evidence of specificity-based effects (Abadie et al., 2021). Specificity effects have not been carefully examined in the context of hyper-binding, so the extent to which older adults' over-reliance on gist-based processing might also play a role in hyper-binding is not known. Taken together, these literatures suggest that age-related declarative memory commission errors may be associated with cognitive processes that are known to decline in the context of cognitive aging. However, the extent to which such errors may result from older adults' explicit decision to report as many items as possible as a sort of compensatory strategy to offset age-related decline in declarative memory or whether these errors occur outside of the explicit adoption of a reduced reporting threshold remains to be explored in future research.

Zooming out, it is possible that combinations of these cognitive factors might be present at any one time to contribute to commission errors in declarative memory. It is important to note that although the false memory, associative memory, and hyper-binding literatures are supportive of the emergence of higher rates of age-related commission errors in declarative memory, and more specifically in the context of recall tasks, some of these paradigms make use of recognition memory tasks. The associative memory literature, in particular, commonly relies on scores from recognition memory probes. The DRM paradigm literature has also made use of recognition memory tasks in addition to recall-based measures. Despite the use of recognition memory tasks in some of the past work on commission errors, converging evidence from the fields of false memory, associative memory, and hyper-binding suggest that commission errors in the context of recall tasks might also occur with fairly high frequency in healthy older adult populations. This is useful to consider given that the emergence of significant declarative memory omission errors is typically not visible until rather late into adulthood, around the mid-fifties (Nyberg et al., 2012).

In addition, the patterns of commission errors that emerge under paradigms employed in these disparate literatures may relate more strongly to some types of commission errors over others, consistent with findings from the everyday action literature (Bailey et al., 2013). It remains to be seen exactly how the cognitive processes underlying the false memory, associative memory, and hyper-binding phenomena may contribute to and help distinguish between different types of commission errors like substitutions or additions. Results of such studies may also reveal important distinctions between neural activity associated with omission and commission errors broadly, and also help to reveal specific neural underpinning of different subtypes (e.g., substitution vs. additions) of commission errors. Such distinctive error profiles may eventually become relevant to the conversation around pathological aging compared to healthy aging. By first understanding error profiles in healthy aging, future research could examine the extent to which error types in declarative memory are sensitive to shifts toward cognitive impairment, in addition to standard free recall composite scores that are often used in used in cognitive assessments for this purpose.

Given the aforementioned reliance on recognition tests in the associative memory literature, there is further reason for additional studies to be centered on commission errors examined in the context of free recall. In the future, researchers may wish to take advantage of available datasets that contain delayed-recall data to re-examine patterns in participant responses for evidence of commission errors. In addition, researchers may also consider designing or modifying existing tasks to elicit higher rates of commission errors and to better characterize individual differences in commission error rates as people age. Using these more targeted paradigms, it is possible that differences in commission errors may also show to be predictive of omission errors in healthy aging. Overall, a more thorough characterization of commission errors in older adults can help to refine the field's understanding of changes in declarative memory with advancing age.
